# A metal artifact reduction method for small field of view CT imaging

**DOI:** 10.1371/journal.pone.0227656

**Published:** 2021-01-14

**Authors:** Seungwon Choi, Seunghyuk Moon, Jongduk Baek

**Affiliations:** School of Integrated Technology and Yonsei Institute of Convergence Technology, Yonsei University, Yeonsu-gu, Incheon, South Korea; Polytechnic University of Marche, ITALY

## Abstract

Several sinogram inpainting based metal artifact reduction (MAR) methods have been proposed to reduce metal artifact in CT imaging. The sinogram inpainting method treats metal trace regions as missing data and estimates the missing information. However, a general assumption with these methods is that data truncation does not occur and that all metal objects still reside within the field-of-view (FOV). These assumptions are usually violated when the FOV is smaller than the object. Thus, existing inpainting based MAR methods are not effective. In this paper, we propose a new MAR method to effectively reduce metal artifact in the presence of data truncation. The main principle of the proposed method involves using a newly synthesized sinogram instead of the originally measured sinogram. The initial reconstruction step involves obtaining a small FOV image with the truncation artifact removed. The final step is to conduct sinogram inpainting based MAR methods, i.e., linear and normalized MAR methods, on the synthesized sinogram from the previous step. The proposed method was verified for extended cardiac-torso simulations, clinical data, and experimental data, and its performance was quantitatively compared with those of previous methods (i.e., linear and normalized MAR methods directly applied to the originally measured sinogram data). The effectiveness of the proposed method was further demonstrated by reducing the residual metal artifact that were present in the reconstructed images obtained using the previous method.

## Introduction

During X-ray computed tomography (CT) imaging, the presence of metallic implants, such as dental fillings, hip prostheses, and orthopedic implants, can introduce metal artifact in the reconstructed images. The high atomic numbers of the metals cause photon starvation, beam hardening, and scatter, which produce severe dark and bright streak artifact in reconstructed CT images [1, 2], thereby degrading the image quality and diagnostic performance.

Several metal artifact reduction (MAR) algorithms have been proposed. In one of the most widely known methods called sinogram inpainting, metal trace regions are determined within the sinogram corrupted by metal objects and replaced with appropriate correction values. Various methods are used to fill in these metal trace regions, such as simple linear interpolation using peripheral values (linear MAR or LMAR) [3] and other techniques based on high-order [4–6], wavelet [7, 8], and prior knowledge [9, 10]. However, these methods are only effective for simple structures and may introduce additional artifact owing to the increase in the estimation errors when applied to images containing complex anatomical structures. The normalized MAR (NMAR) [11] method can reduce estimation errors in metal trace regions using a prior image, and is usually acquired from the initially corrected images from LMAR. Note that the prior image is not equivalent to prior knowledge but is obtained from the data itself. Although normalizing the sinogram by a prior sinogram can reduce the interpolation errors within the metal trace regions, the performance of the NMAR is affected by the quality of the prior image. If the original image contains severe residual artifact, the prior image also contains severe errors that degrade the performance of the NMAR method.

To reduce the metal artifact more effectively, several iterative approaches have been proposed [12–15]. Specifically, various regularization terms such as total variation [12, 13] or quadratic smoothness function [14], have been adapted to handle the ill-posed conditions in MAR. A material decomposition method using spectral CT and MAR through a penalized maximum likelihood iterative reconstruction has also been proposed [15]. Compared to the sinogram inpainting methods, the iterative MAR methods are robust to noise but are still prone to issues with computation time.

Recently, several deep learning based MARs have been proposed. A simple image domain approach is to train a convolutional neural network (CNN) to reduce the residual errors in the NMAR image, where the training data pair is composed of the NMAR image (input) and a reference image (target) [16]. In [17], input data are composed of 3-channel uncorrected original image, beam hardening corrected image, and linear MAR images, and the correction performance is further improved by fully utilizing information from the 3-channel input data during network training. In the sinogram domain approach, the CNN is trained to estimate the missing data in the metal trace regions using two simple hidden layers [18] and conditional generative adversarial network (cGAN) [19], which are more effective than traditional interpolation based approaches. A method that utilizes data from both domains is presented in [20], where the benefits of the CNN in each domain are combined to improve the performance of the MAR method. Although deep learning based approaches have shown impressive results in MAR, they require a large amount of data for network training.

In the typical sinogram inpainting method [3–8, 11], the metal is segmented from the uncorrected image and then forward projected to identify the metal trace region. While inpainting based methods are effective for MAR, it is assumed that object truncation does not occur and that all metal objects exist within the field-of-view (FOV). However, when the FOV is small and multiple metal objects are present outside this FOV (such as in dental CT imaging) or when the metal object is not completely within the FOV (such as bone biopsy needles), the assumptions of the inpainting based MAR algorithms are violated. When metal objects are present outside of FOV, the reconstructed image within the FOV cannot be used to locate them; thus, the metal trace region from the metal outside the FOV can not be identified correctly. In addition, the truncated projection data still contain the object information outside the FOV. Thus, the energy level between the forward projection of the prior image acquired from the truncated corrected image and the truncated projection data are not matched. As a result, the NMAR method is not effective for such applications.

In our previous work, we proposed a MAR method based on multiple prior images using a recursive active contour segmentation [21] scheme; however, when data truncation occurs, the residual artifact of the initial MAR process can cause severe distortions, thus making it difficult to apply this method. In this work, we propose a new method for MAR in small FOV imaging with data truncation. The proposed method achieves MAR using a sinogram of the small FOV image obtained through forward projection of an uncorrected image with metal artifact that has only truncation corrections. The proposed method is validated using simulated extended cardiac-torso (XCAT) data, clinical data, and experimental cylinder phantom data containing metal implants outside the small FOV. The performance of the proposed method is compared with those of previous methods (i.e., LMAR and NMAR directly applied to the originally measured sinogram data). The normalized mean-squared error (NMSE) and structure similarity (SSIM) parameters were used to quantitatively evaluate and compare these methods.

## Methods

### Proposed method

**Fig 1:**
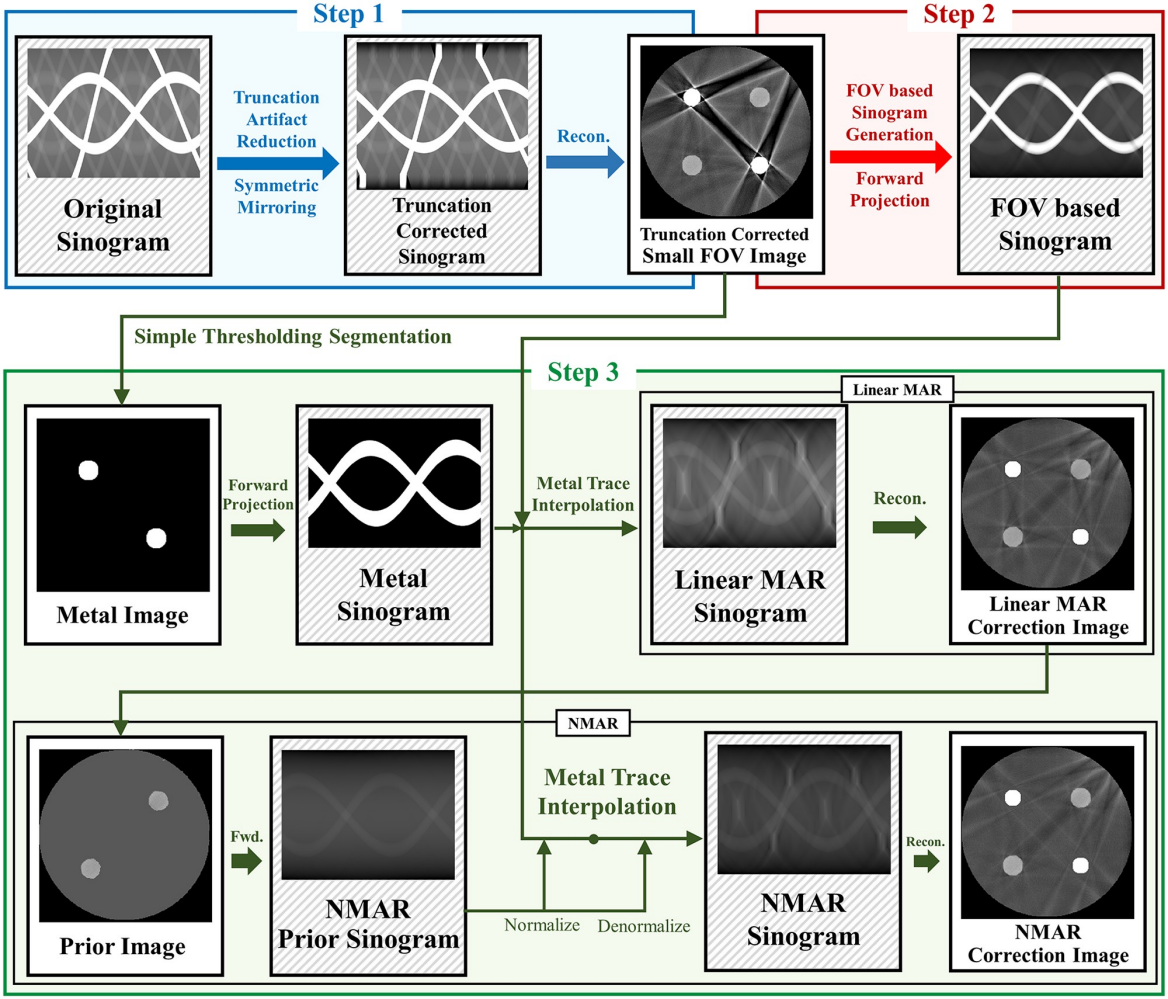
Diagram of the proposed method. The proposed method consists of three steps. Step 1 shows truncation artifact correction. Step 2 shows small FOV sinogram synthesizing. Step 3 shows sinogram inpainting based MAR method.

In CT imaging, if the scanning area is smaller than the object, truncations occur in the projection measurements, which cause truncation artifact. These truncated projection measurements are then reconstructed as a small FOV image. In this case, if the metal objects are present outside the small FOV, metal artifacts within the small FOV region cannot be corrected effectively by the traditional LMAR and NMAR methods. In the proposed method, we first remove the truncation artifact to reduce the influence of objects outside the small FOV and then reduce the metal artifact. A small FOV image with only truncation artifact correction is obtained using the truncation artifact reduction method. After generating a sinogram by forward projection of this small FOV image, the generated sinogram is processed using sinogram inpainting based MAR instead of the originally measured uncorrected sinogram. The overall scheme of the proposed method is depicted in Fig 1, and the details of each step are as follows.

Step 1: Truncation artifact were corrected and the small FOV image was obtained. The originally measured sinogram has missing information outside the small FOV due to projection truncation; hence, truncation correction is necessary to reduce the truncation artifact in small FOV images. To reduce truncation artifact, we used the symmetric mirroring method [22] which estimates the projection data near the truncated region in the sinogram domain by assuming that the data outside the small FOV has a similar symmetry to the data inside the small FOV. After step 1, no other truncations occur in the subsequent processing steps.
  To estimate the projection data of the *N_s_* pixels within the truncated region, we symmetrically flip the inner sinogram by *N_ext_* pixels (conventional CT geometry uses 300 and dental CT geometry uses 150 pixels in this study) and multiply them with a weighting function (a cosine function from 0-90° in this study). The weighting function for the *N*-th pixel is calculated as
  $$\begin{array}{c} Weightingfunction(N)=\{\begin{array}{cc} cos(\frac{N_{ext}-N}{N_{ext}}*\frac{\pi }{2}) & N=0,1,2\ldots N_{ext}-1\\ 1 & N=N_{ext},N_{ext}+1,\ldots N_{ext}+N_{s}-1\\ cos(\frac{N-N_{s}-N_{ext}+1}{N_{ext}}*\frac{\pi }{2}) & N=N_{ext}+N_{s},\ldots ,2N_{ext}+N_{s}-1 \end{array} \end{array}$$ (1)
Step 2: The synthesized sinogram was acquired by forward projection of the small FOV image after the truncation artifact correction. Note that the forward projection in this step was conducted using a five-times finely sampled the original detector size to reduce discretization errors.
Step 3: The synthesized sinogram in step 2 is used for sinogram inpainting based MAR. In this study, both LMAR [3] and NMAR [11] are used. For both MAR methods, to find the metal trace region in the sinogram domain, we segmented the metal from a small FOV reconstruction image using a simple thresholding and then performed a forward projection to find the metal sinogram. Then, LMAR was conducted within the metal trace region. The image used in NMAR is generated using LMAR result. Using simple thresholding, the bone pixels are used as is, while the soft tissue pixels are filled with a single value that is equal to the average value of the soft tissue that is not effected by metal artifact. The prior sinogram is generated by forward projection of a prior image. The synthesized sinogram from the small FOV image is normalized by dividing with the prior sinogram. The normalized sinogram is interpolated and then denormalized by the prior sinogram. All interpolations used in this study were linear in the radial direction. The actual code of the proposed method is available in S1 File.

### XCAT data

**Fig 2:**
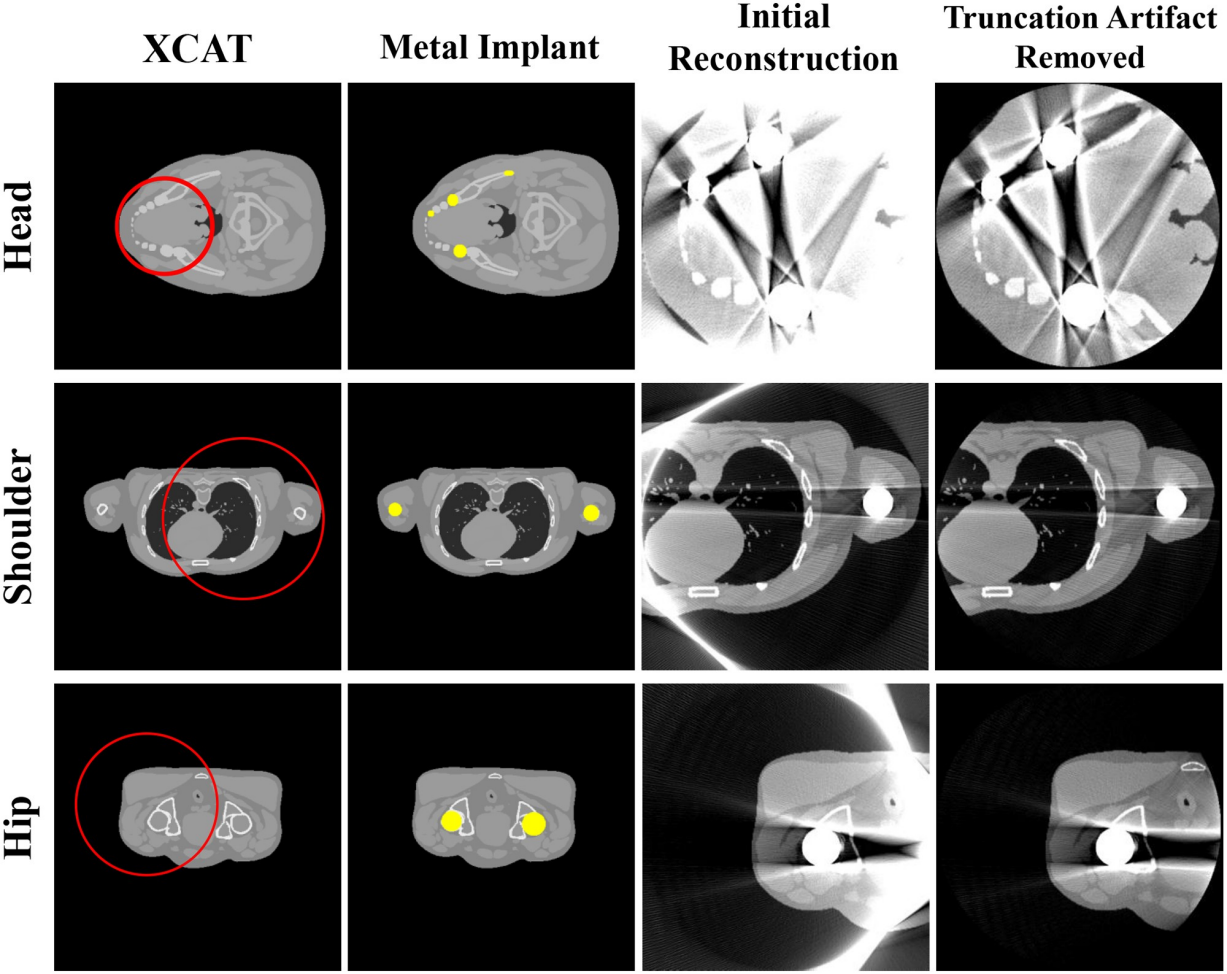
Representative images of the XCAT data. Each row corresponds to a different part of the body. Each column represents the original XCAT image, metal implant, reconstructed image, and reconstructed image with truncation artifact correction. The red circles in the images represent the small FOV for each case. Display window width/window level = 2000 HU/0 HU.

To validate the proposed method, the human head, shoulders, and hips were simulated using the XCAT phantom images developed by Segars [23]. A metal implant was created as a binary image by setting the position and shape similar to where the actual metal implant would be present in the human body, after which the metal image was inserted into the original XCAT image. Fig 2 shows the phantom and first reconstruction results.

**Fig 3:**
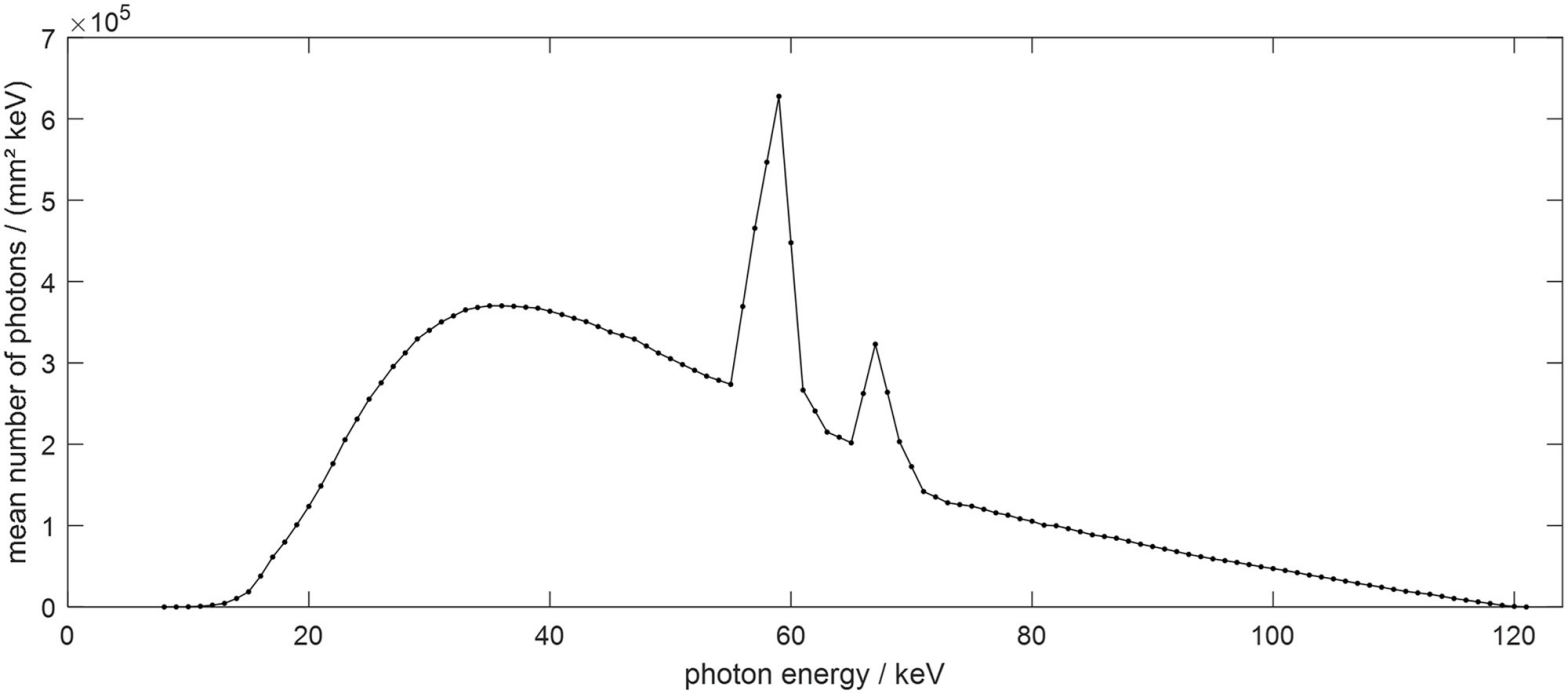
Siemens X-ray spectrum at 120 kVp.

In the simulations, the projection data were acquired using a polychromatic energy spectrum:

$$\begin{array}{c} I=I_{0}\int \Omega \left(E\right)\exp\left(-\int \mu_{E,S}dS\right)dE, \end{array}$$ (2)

where *I* represents the transmitted intensity, *I*_0_ is the incident intensity of the polychromatic energy, *μ*_*E*,*S*_ represents the linear attenuation coefficient of the object for each energy, and Ω(*E*) denotes the incident X-ray spectrum for each energy. The polychromatic projection data were acquired with a 120 kVp tube voltage using the Siemens X-ray spectrum [24]. Tungsten was used as the X-ray shooting anode material and note that an additional beam filter such as a bow tie filter was not used. A detailed plot of X-ray spectrum is shown in Fig 3. In the XCAT simulations, water, bone, and metal are the main components of the object; thus Eq 2 can be expressed as

$$\begin{array}{c} I=I_{0}\int \Omega \left(E\right)\exp\left(-\mu_{w,E}d_{w}-\mu_{b,E}d_{b}-\mu_{m,E}d_{m}\right)dE, \end{array}$$ (3)

where, *μ_w,E_*, *μ_b,E_*, and *μ_m,E_* are the attenuation coefficients of water, bone, and metal, respectively, at the specific energy band *E* and were obtained from the NIST X-ray attenuation database [25] and *d_w_*, *d_b_*, and *d_m_* are the path lengths of water, bone, and metal, respectively. In this study, we used gold as the metal. The transmitted intensity then follows the Poisson distribution:

$$\begin{array}{c} y∼\text{Poisson}\left\{I+r\right\}, \end{array}$$ (4)

where r is the mean number of the background events and readout noise variance [26–28]. Thus, the noisy polychromatic projection *p* is expressed as

$$\begin{array}{c} p=-\ln(\frac{y}{I_{0}}). \end{array}$$ (5)

To simulate a polychromatic projection, it is necessary to know the composition of the materials in each pixel; hence, the XCAT phantom image was divided into individual images according to the material types. First, the inserted metal artifact was excluded by simple thresholding, and the XCAT data were divided into bone and soft tissue images using the pixel values from the remaining images. A simple thresholding step was sufficient to segment the metal, soft tissue, and bone images since each pixel of the XCAT data did not contain a mixture of different tissues. The corresponding attenuation values and polychromatic spectra from the material specific images were then applied to obtain the polychromatic projections.

Forward projection and reconstruction were performed using the TIGRE: Matlab-GPU Toolbox [29]. The detailed parameters for XCAT and the subsequent simulations are summarized in Table 1. Two different fan-beam CT geometries were used to confirm the feasibility of the algorithm for both conventional and dental CT systems. The reconstructed image size was 512 x 512 pixels, and the truncations occurred with the corresponding geometries. The diameter for the small FOV was the same as that of the reconstructed image. Each detector cell was expected to receive **1**0^5^ photons in the case of blank scan and each measurement followed the Poisson distribution. In the polychromatic projection generation, we used 12 source energies in the range of 10-120keV with a step size of 10keV. To verify the effectiveness of the algorithm, the initial object was cropped to fit the small FOV size to prevent truncation errors, after which the reference image was reconstructed from the generated polychromatic projection data.

**Table 1:** Parameters for data acquisition and reconstruction for both the simulations and the experiments.

Parameters	Conventional CT Geometry	Dental CT Geometry
Source to iso-center distance	700 mm	500 mm
Detector to iso-center distance	500 mm	200 mm
Number of views	720 view	720 view
Detector cell size	0.388 × 0.388 mm^2^	0.388 × 0.388 mm^2^
Detector number	1024	512
Reconstructed image size	358.4 × 358.4 mm^2^	143.36 × 143.36 mm^2^
Reconstructed matrix size	512 × 512	512 × 512
Reconstructed pixel size	0.7 × 0.7 mm^2^	0.28 × 0.28 mm^2^

### Clinical data

**Fig 4:**
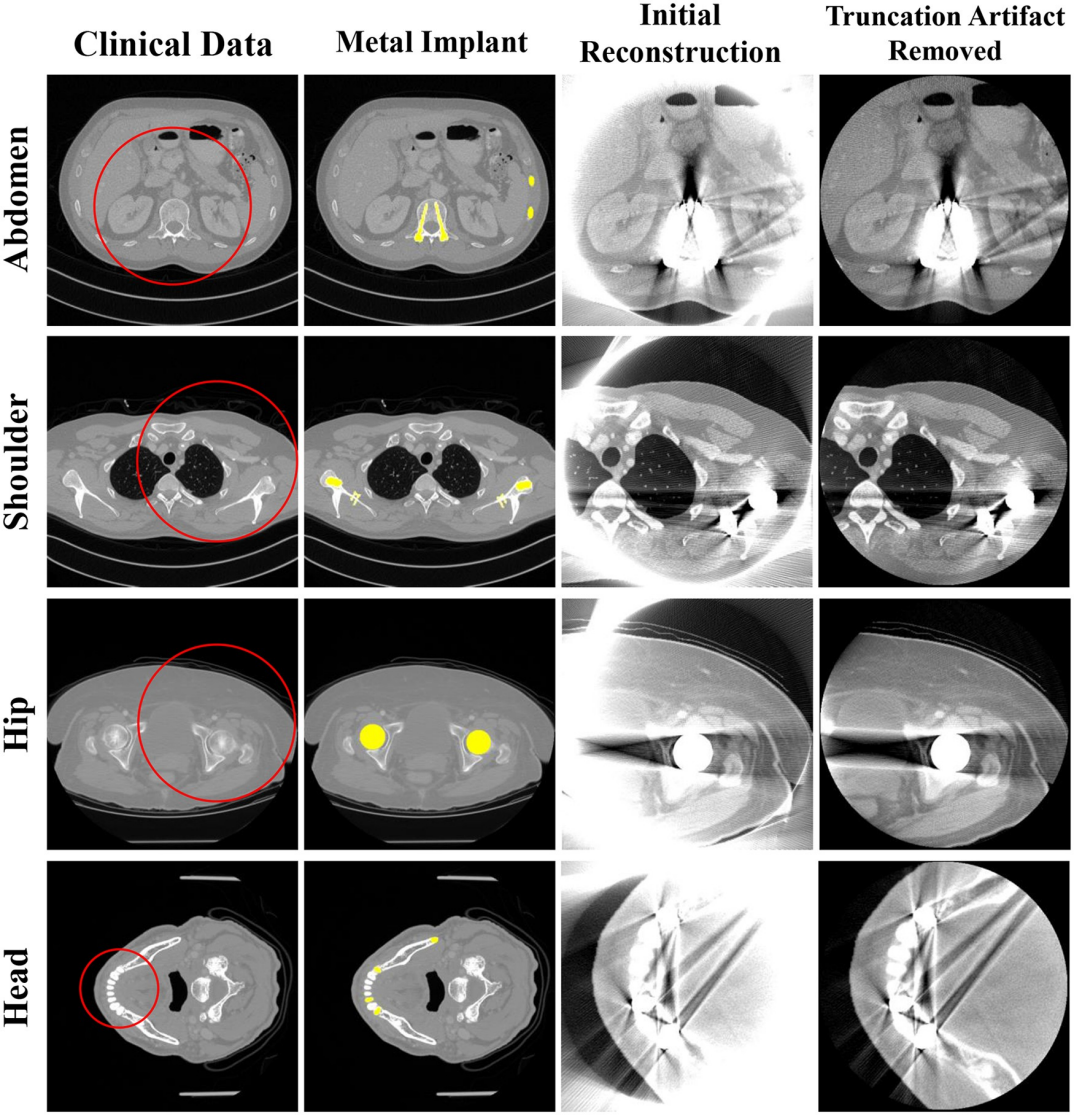
Representative images of the clinical data. Each row corresponds to a different part of the body. Each column represents the original clinical image, metal implant, reconstructed image, and reconstructed image with truncation artifact correction. The red circles in the images represent the small FOV for each case. Display window width/window level = 2000 HU/0 HU.

A simulation study was conducted using clinical data to evaluate the performance of the proposed method. We used reconstructed image data from the “2016 Low-dose CT Grand Challenge” dataset [30] and the cancer imaging archive [31]. Abdomen, shoulder, hip, and head images were selected as the former three had geometries similar to a general conventional CT scan (Fig 4) and the head image had a geometry similar to a dental CT scan (Table 1). Metal implants were inserted into the clinical reconstruction images to generate metal artifact. By following the same procedure to generate the polychromatic projection data as in the XCAT simulations, we created binary images of the metal implants similar to those in a real human body and replaced the corresponding parts in the clinical data. The metal artifact inserted images were reconstructed using the generated polychromatic projection data, and the proposed algorithm was applied to reduce the metal artifact.

Unlike the XCAT data, the clinical images are not classified precisely by pixel values for each material in the target. Therefore, to generate the polychromatic projections, the pixel values were provided as those for a mixture of materials by considering the ratio of the each components. After excluding metal area, the soft tissue is identified when the *i^th^* pixel value *i^i^* is less than threshold *T*_1_(i.e., 80 HU), the bone is identified when it was larger than the threshold *T*_2_ (i.e., 660 HU), and when the value of the pixel was between *T*_1_ and *T*_2_, it was identified as the mixture of water and bone; the mixture ratio is obtained using a soft threshold based weighting method [32]:

$$\begin{array}{c} w(x^{i})=\{\begin{array}{cc} 0 & x^{i}<=T_{1}\\ 1 & x^{i}>=T_{2}\\ \frac{x^{i}-T_{1}}{T_{2}-T_{1}} & T_{1}<x^{i}<T_{2} \end{array}, \end{array}$$ (6)

where *w*(*x^i^*) is the *i^th^* pixel weighting. Hence, the *i^th^* pixel components of bone and water ($x_{b}^{i}$ and $x_{w}^{i}$, respectively) are expressed as

$$\begin{array}{c} x_{b}^{i}=w\left(x^{i}\right)x^{i}, \end{array}$$ (7)

$$\begin{array}{c} x_{w}^{i}=\left(1-w\left(x^{i}\right)\right)x^{i}. \end{array}$$ (8)

For the bone, water, and metal images, the attenuation coefficient for each energy band was applied according to the spectrum at 120 kVp. We used the same method to generate the polychromatic projections as that described in the previous section for the XCAT data. To compare the performance of the algorithm for the clinical data, a reference image was created using the same procedure as that for generating the reference XCAT data.

### Experiments

**Fig 5:**
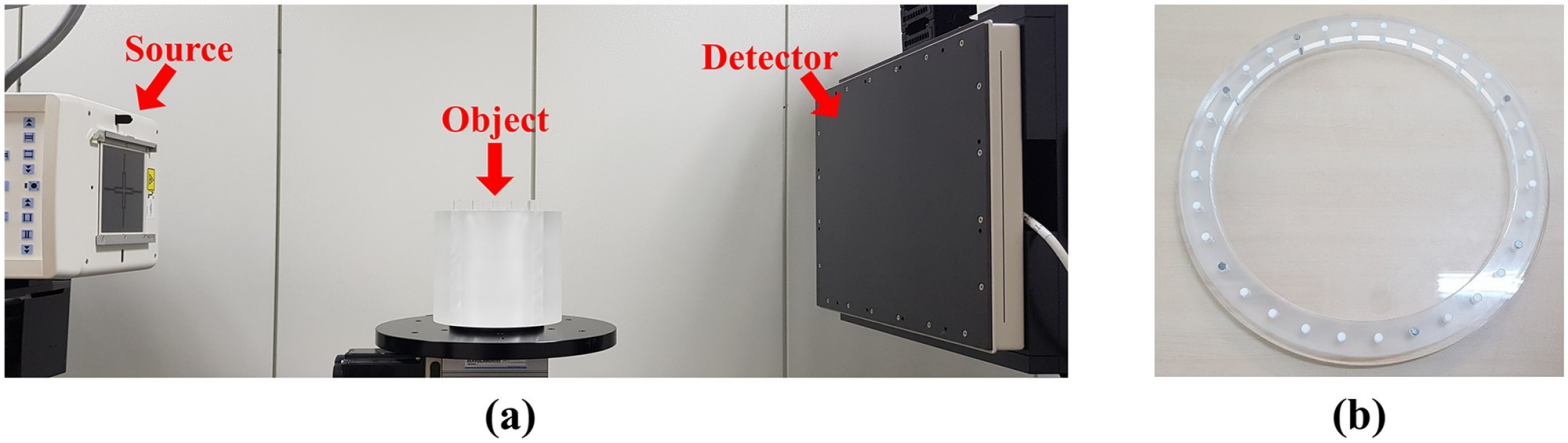
Experimental setup. (a) Experimental benchtop system and (b) semi-top view of the disk phantom.

For the experimental data acquisition, we used the benchtop system as shown in Fig 5(a). The geometry parameters for the benchtop system were identical to those of the conventional CT geometry in the XCAT and clinical data (Table 1). We conducted the experiments using a disk phantom of 25 cm in diameter with 32 cylinders of 5 mm diameter inserted along its perimeter (Fig 5(b)). To examine the robustness of the proposed method, we examined three cases where the number of metal objects outside the small FOV increased from one to three. Note that the disk phantom contained three metal objects inside the small FOV.

We obtained the projection data using the benchtop CT system comprising of a generator (Indico 100, CPI Communication and Medical Products Division, Georgetown Ontario, Canada), a tungsten target X-ray source with a 0.6 × 0.6 mm^2^ focal spot (Varian G-1592, Varian X-ray Products, Salt Lake City, UT), and a 300 × 400 mm^2^ flat panel detector (PaxScan 4030CB, Varian Medical Systems, Salt Lake City, UT). The projection data for the disk phantom were obtained at 90 kVp and 6 mA and Feldkamp-Davis-Kress reconstruction was performed on 512 × 512 matrices with a pixel size of 0.7 × 0.7 mm^2^.

### Image quality evaluation

The NMSE and SSIM [33] were used to quantitatively evaluate and compare the image qualities of the previous and proposed methods. The NMSE represents the difference in value between the two images, and the SSIM indicates how similar the structures of the two images are. The NMSE is defined as

$$\begin{array}{c} NMSE=\frac{1}{N}\frac{\sum_{k=1}^{N}{\left(f\left(k\right)-f_{ref}\left(k\right)\right)}^{2}}{\bar{f(k)}\;\bar{f_{ref}\left(k\right)}}, \end{array}$$ (9)

$$\begin{array}{c} \bar{f(k)}=\frac{1}{N}\sum_{k=1}^{N}f\left(k\right)\;\;\;and\;\;\;\bar{f_{ref}\left(k\right)}=\frac{1}{N}\sum_{k=1}^{N}f_{ref}\left(k\right), \end{array}$$ (10)

where *f*(*k*) and *f*_*ref*_(*k*) represent the intensities of the target and reference images at pixel location *k*, respectively; *N* is the number of image pixels; and $\bar{f(k)}$ and $\bar{f_{ref}\left(k\right)}$ denote the average intensities of the target and reference images, respectively.

The SSIM is defined as

$$\begin{array}{c} SSIM(A,B)=\frac{\left(2\mu_{A}\mu_{B}+C_{1}\right)\left(2\sigma_{AB}+C_{2}\right)}{\left({\mu_{A}}^{2}+{\mu_{B}}^{2}+C_{1}\right)\left({\sigma_{A}}^{2}+{\sigma_{B}}^{2}+C_{2}\right)}, \end{array}$$ (11)

where *μ*_*A*_, *σ*_*A*_, *μ*_*B*_, and *σ*_*B*_ represent the average intensities and standard deviations of image *A* and *B*, respectively; *σ*_*AB*_ is the covariance between the images; and *C*_1_ and *C*_2_ denote the coefficients for the SSIM calculations and range from 0 to 1. The NMSE and SSIM were calculated between the reference image and the corrected MAR images.

## Results

### XCAT data results

**Fig 6:**
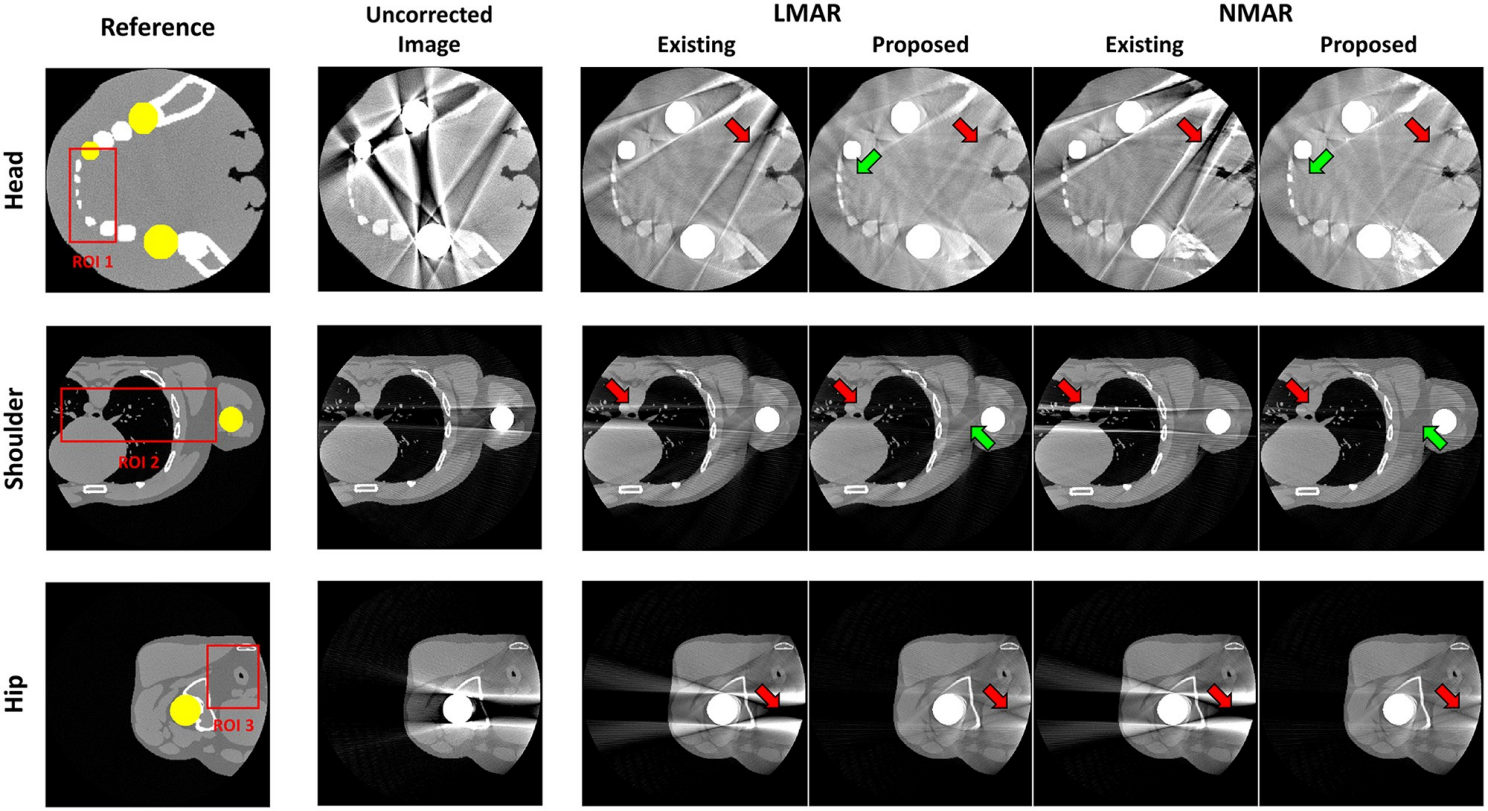
Results using XCAT data. Each row corresponds to a different part of the body. Each column represents the reference image, uncorrected metal artifact image (corrected for only truncation artifact), and LMAR and NMAR result images using the previous and proposed methods. Display window width/window level = 2000 HU/0 HU.

Fig 6 shows the results of the previous and proposed LMAR and NMAR methods from XCAT data. Each row corresponds to the head, shoulder, and hip region. The leftmost column shows the reference image for each case, which is an image obtained by creating an object without truncation and the metal. In each reference image, the yellow area represents the metal object region, and the red box represents the area where the NMSE and SSIM are measured for image quality evaluation. The resulting images show that both MAR methods can reduce the metal artifact. However, as indicated by the red arrows, the proposed method provided superior performance for both LMAR and NMAR compared to the previous method. The metal artifact produced by the metals inside the small FOV were effectively reduced by both the previous and proposed methods, as shown in the head image. However, the previous LMAR and NMAR methods were not effective at removing the artifact produced by the external metal objects, although the proposed method is still effective for reducing them. It can also be seen that the NMAR reduced metal artifact more effectively than the LMAR, as indicated by the green arrows in Fig 6.

**Fig 7:**
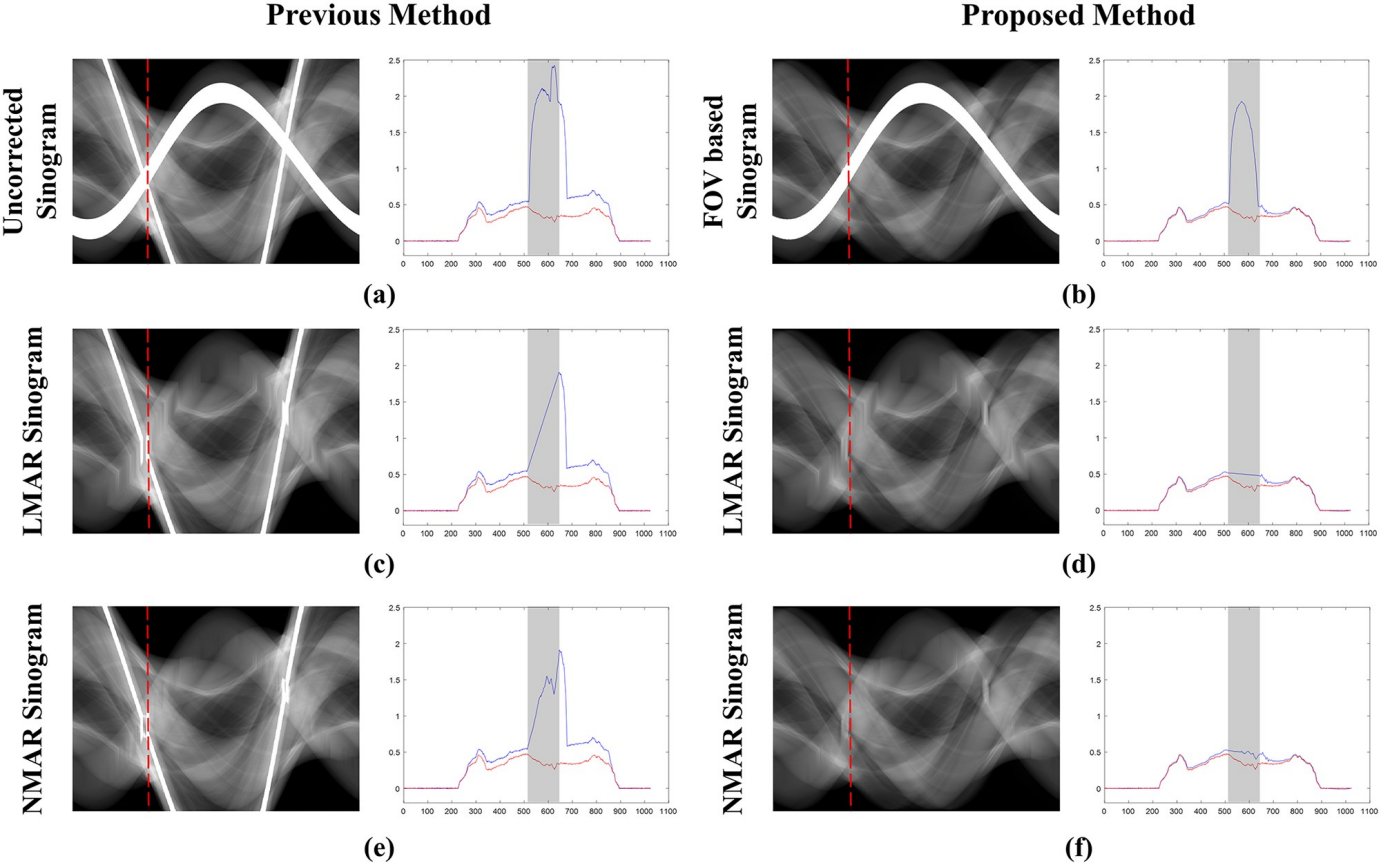
Sinograms and central profiles (189th view) of the XCAT shoulder results. (a) Originally measured uncorrected sinogram, (b) small FOV based sinogram, (c,e) sinograms with the previous LMAR and NMAR methods, and (d,f) sinograms with the proposed LMAR and NMAR methods. The red dotted lines in the sinogram images represent the central profiles at the 189th view, and the gray shading in the central profiles represent the metal trace regions. The blue lines in the plots represent the sinogram values at the 189th view, and the red lines represent the reference sinogram values at the 189th view.

Fig 7 shows the sinogram used for the XCAT shoulder images and projection data in the 189th view, along with the resulting sinograms from the previous and proposed LMAR and NMAR methods. In the central profile graph, the blue line is the value of each resulting sinogram, and the red line is the value of the reference sinogram. The gray shaded region of the plot indicates the metal trace region where interpolation was conducted. It is clear that the proposed method estimated the reference sinogram better than the previous method as the former uses the sinogram created through forward projection of the small FOV in Fig 7(b) instead of the originally measured sinogram. Thus, the value outside the metal trace region matches well with that in the reference sinogram. In particular, it can be observed that with the previous method, the sinogram was corrupted by the external metal trace during interpolation, and this was effectively prevented in the proposed method. In addition, the previous NMAR method amplified the estimation error within the metal trace region when the area corrupted by the metal object outside the small FOV was normalized and denormalized; this adversely affected the performance of the previous LMAR method compared to that of the proposed one.

Table 2 summarizes the NMSE and SSIM values for LMAR and NMAR for the previous and proposed methods. The region of interest (ROI) in the reference images of the head, shoulder, and hip were used in the comparison. The NMSE and SSIM values show that the proposed method performs better than the previous method. In addition, the NMAR performs better than the LMAR for the proposed method.

**Table 2:** NMSE and SSIM results with XCAT images.

		NMSE		SSIM	
		Previous	Proposed	Previous	Proposed
ROI 1	LMAR	0.0458	0.0380	0.8606	0.8763
	NMAR	0.0602	0.0326	0.8313	0.8810
ROI 2	LMAR	0.0885	0.0432	0.6294	0.7222
	NMAR	0.1961	0.0348	0.6043	0.7436
ROI 3	LMAR	0.2191	0.0325	0.7160	0.8365
	NMAR	1.3586	0.0317	0.5980	0.8373

### Clinical data results

**Fig 8:**
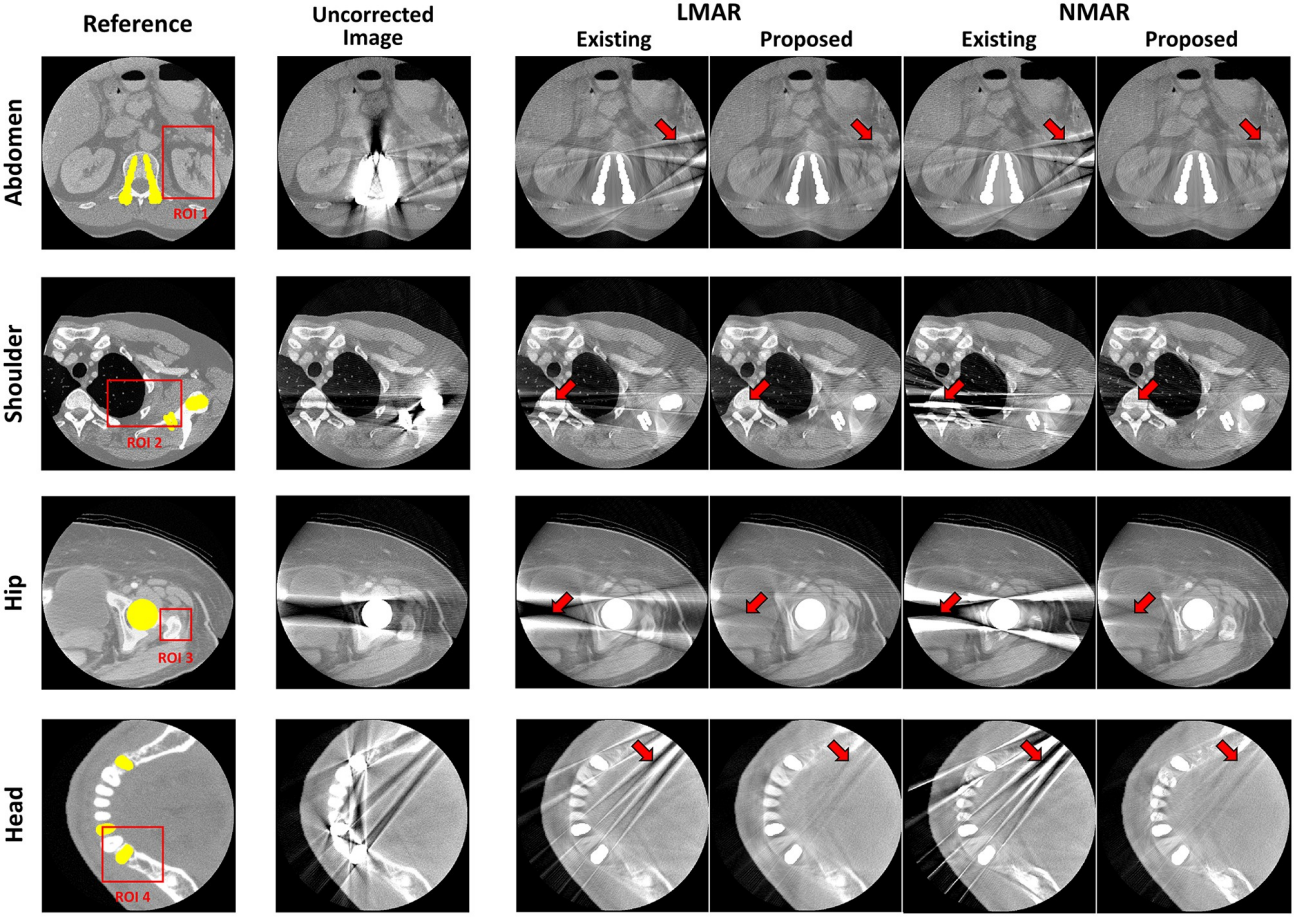
Results using clinical data. Each row corresponds to a different part of the body. Each column represents the reference image, uncorrected metal artifact image (corrected only truncation artifact), and LMAR and NMAR result images using the previous and proposed methods. Display window width/window level = 2000 HU/0 HU.

Fig 8 presents the result images after applying the previous and proposed LMAR and NMAR methods to the clinical data of the abdomen, shoulder, hip, and head. The leftmost column is the reference image for each case, and the area of the metal object is marked in yellow. The red box shows the ROI for quantitative comparisons of the image quality. The next two images show the LMAR results obtained using the previous method (left) and proposed method (right). The last two images are the NMAR results for the previous method (left) and proposed method (right). The results show that all methods reduced the metal artifact compared to the uncorrected metal artifact images. However, as indicated by the red arrows, the residual artifact effectively removed by the proposed method still remain after applying the previous method.

Table 3 summarizes the NMSE and SSIM values for the clinical data for the abdomen, shoulder, and head. The results indicate that the proposed method performs better than the previous method for all cases.

**Table 3:** NMSE and SSIM results with clinical images.

		NMSE		SSIM	
		Previous	Proposed	Previous	Proposed
ROI 1	LMAR	0.0815	0.0339	0.8413	0.9090
	NMAR	0.1128	0.0286	0.8304	0.9162
ROI 2	LMAR	0.1904	0.1632	0.7279	0.7975
	NMAR	0.3824	0.1504	0.6335	0.7986
ROI 3	LMAR	0.0597	0.0350	0.8800	0.9298
	NMAR	0.1517	0.0298	0.7423	0.9407
ROI 4	LMAR	0.0479	0.0442	0.9040	0.9154
	NMAR	0.0459	0.0339	0.8981	0.9155

To validate the proposed method for various clinical data, additional simulations were performed using clinical data, as given in the Supporting Information. These additional simulations were performed on the leg, pelvis, abdomen, lung, spine, shoulder, brain, and orbit regions. The reference and metal inserted images for these simulations are available in S1 Fig, the results of applying the proposed method are given in S2 Fig, and the NMSE and SSIM results are presented in S1 Table.

### Experimental results

**Fig 9:**
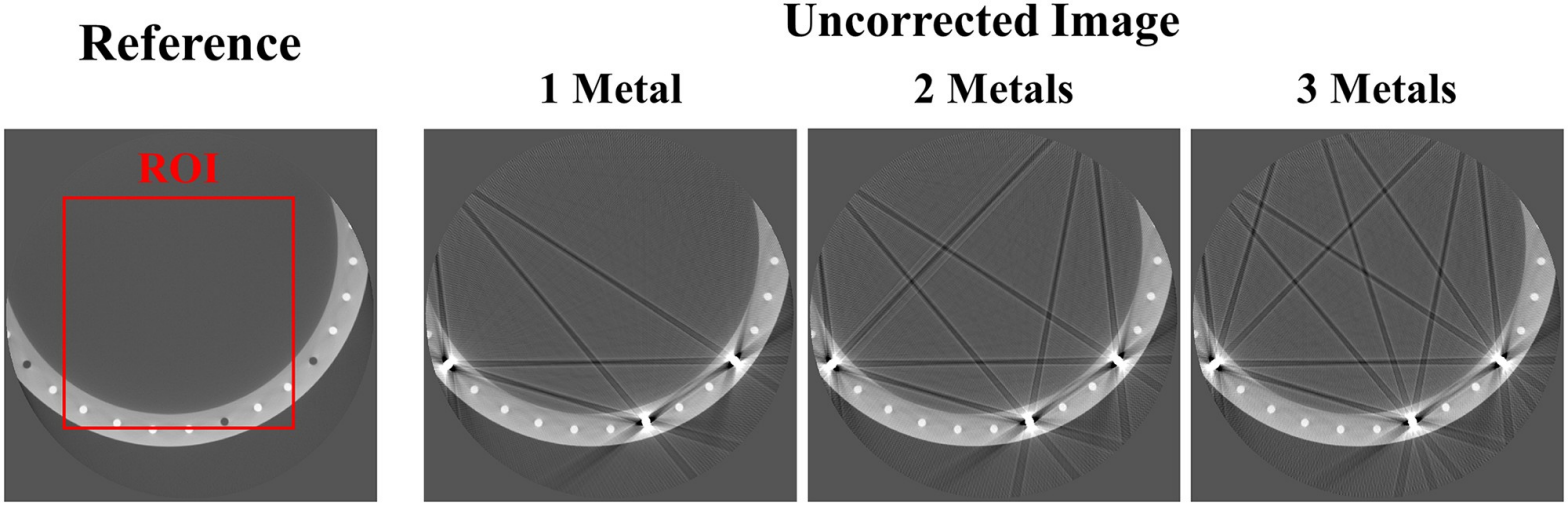
Experimental data. Reference image and uncorrected metal artifact images (corrected for only truncation artifact) using the disk phantom experimental data. The red box represents the ROI which the NMSE and SSIM were computed. Display window width/window level = 3000 HU/-500 HU.

**Fig 10:**
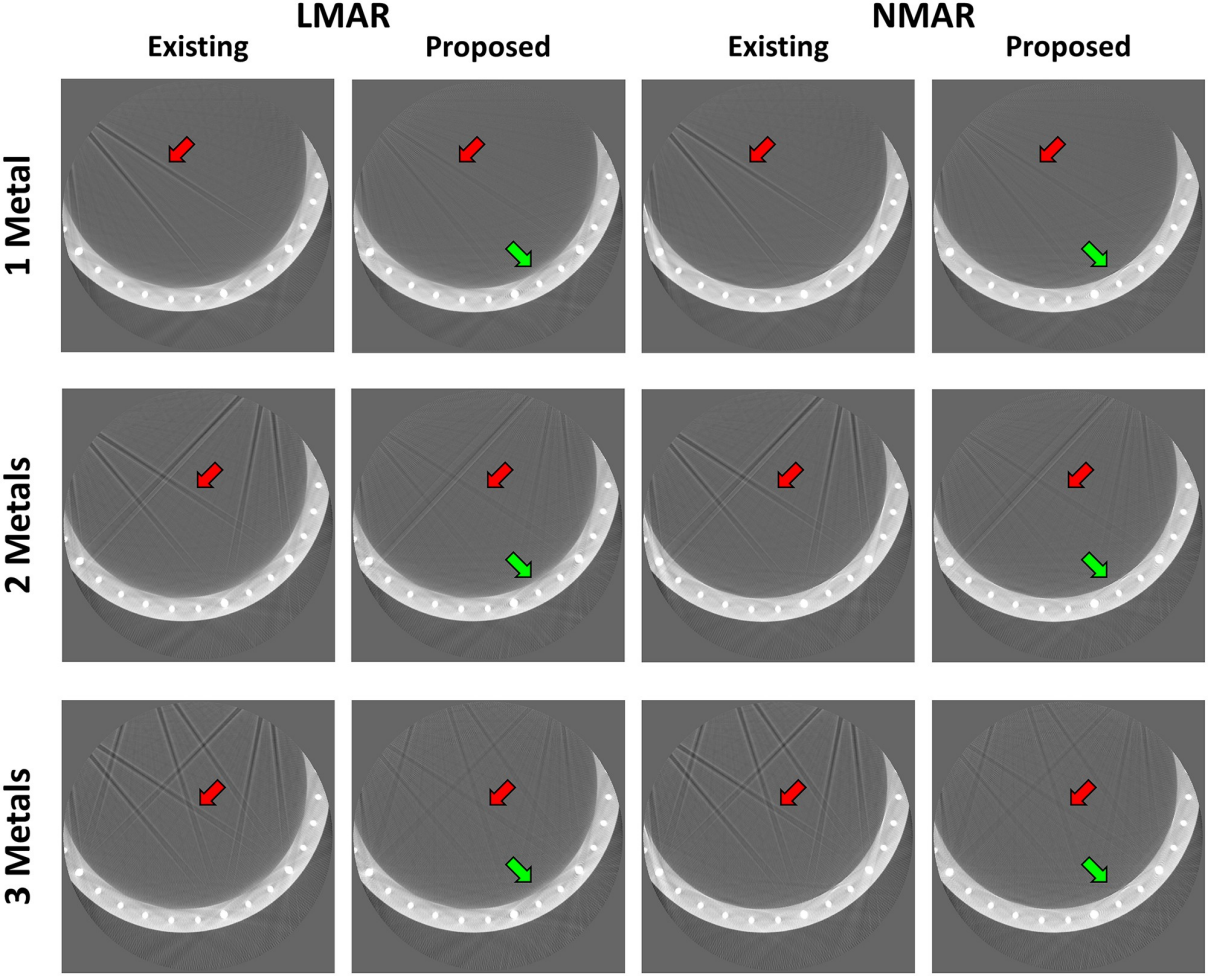
Results using disk phantom experimental data. Each row corresponds to the number of metal objects outside the small FOV. Each column represents the LMAR and NMAR result images using the previous and proposed methods. Display window width/window level = 2500 HU/-750 HU.

Fig 9 shows the original disk phantom, where the number of metals inside the small FOV was fixed as three and number of metals outside the small FOV increased from one to three. The MAR results in Fig 10 are observed to be better than those of the uncorrected images in all cases. However, as indicated by the red arrows, dark streak artifact still remains in the MAR images obtained with the previous method. Moreover, the image distortions became more severe as the number of metal objects outside the small FOV increased. On the other hand, as indicated by the red arrows, the metal artifact were effectively reduced in the MAR images obtained with the proposed method. Although the previous MAR method reduced the streaks caused by the metal artifact inside the small FOV, these residual streak artifact still remained because the artifact produced by the metal objects outside the small FOV were difficult to remove. However, with the proposed method, the small FOV based sinogram mitigated the effects of the external metal trajectories, thereby reducing the residual metal artifact more effectively. It is also observed that the NMAR was more effective at recovering the boundary of the cylinder phantom than the LMAR, as indicated by the green arrows in Fig 10. The quantitative assessments via NMSE and SSIM, given in Table 4, confirmed our observations. The reference and result image data of the XCAT data, clinical data, and experiments are available in S1 Dataset.

**Table 4:** NMSE and SSIM results with experimental images.

		NMSE		SSIM	
		Previous	Proposed	Previous	Proposed
1 Metal	LMAR	0.2031	0.1767	0.8142	0.8338
	NMAR	0.1604	0.1304	0.8184	0.8442
2 Metals	LMAR	0.2447	0.1997	0.7388	0.7754
	NMAR	0.2099	0.1653	0.7397	0.7819
3 Metals	LMAR	0.2702	0.2062	0.7048	0.7554
	NMAR	0.2357	0.1703	0.7089	0.7612

## Discussion and conclusion

Using precise values between the metal trace regions is critical for sinogram inpainting based MAR methods to estimate the missing data in this region. However, when metal objects are present outside the small FOV, the neighboring values of the interpolated metal traces are corrupted, which introduce more interpolation errors and result in residual artifact after MAR. Furthermore, when using a sinogram inpainting method with a prior sinogram, like the NMAR, for small FOV imaging, the level of the originally measured sinogram does not match the inside of the small FOV image, which can introduce additional artifact after MAR. In the proposed method, synthesized projection data are acquired by truncation corrected small FOV images, which are then used instead of the originally measured sinograms; this minimizes the effects of the metal objects present outside the small FOV during the inpainting procedure.

We validated the proposed algorithm for various clinical data.However, owing to limited access to real clinical data, we could not include the results from real clinical data with metal inserts. Instead, we validated the proposed method using clinical images artificially by inserting the effects of such metals [17, 34, 35]. In the experiment section, real data obtained from scanning metal inserted phantom was used to validate the proposed method.

In this study, only the LMAR and NMAR were used to evaluate the results. Since the proposed method uses a small FOV based sinogram instead of the originally measured one, it can be applied to other sinogram inpainting methods, such as the metal deletion technique [36] and total variation based MAR [12, 13]. In addition, the proposed method proceeds by forward projection of the first reconstructed image rather than the originally measured sinogram, which means that it starts from the image domain; thus, the originally measured sinogram is not needed. Therefore, given the data acquisition geometry, the proposed method can be used to reduce metal artifact in CT images.

In conclusion, we propose a small FOV based MAR method that considers the effects of data truncation. The proposed method proceeds via forward projection of only truncation corrected small FOV images instead of the original sinograms for MAR. The results confirm that LMAR and NMAR with the proposed method can reduce artifact generated by metal objects, which are located both inside and outside the small FOV, that cannot be removed effectively by the previous methods.

## Supporting information

S1 File Actual code of proposed method. Actual code includes main code, truncation reduction function, LMAR, and NMAR function code.
(ZIP)

S1 Fig Supplementary simulation reference image. Representative images of additional simulations. Original clinical and metal implant images. Display window width/window level = 2500HU/250HU.
(TIF)

S2 Fig Supplementary simulation results image. Results with additional clinical data simulation. Red boxes are the ROI of each case. Display window width/window level = 2500HU/250HU.
(TIF)

S1 Table Supplementary table for NMSE and SSIM experiments. Quantitative evaluations of the additional clinical data simulations. NMSE and SSIM for each ROI.
(DOCX)

S1 Dataset Reference images and result images for the data used in the study. (ZIP)
