# Supplementary figures and images for: A metal artifact reduction method for small field of view CT imaging
Source: PLoS One. 2021 Jan 14;16(1):e0227656. doi: 10.1371/journal.pone.0227656 (PMC7808647; doi:10.1371/journal.pone.0227656)

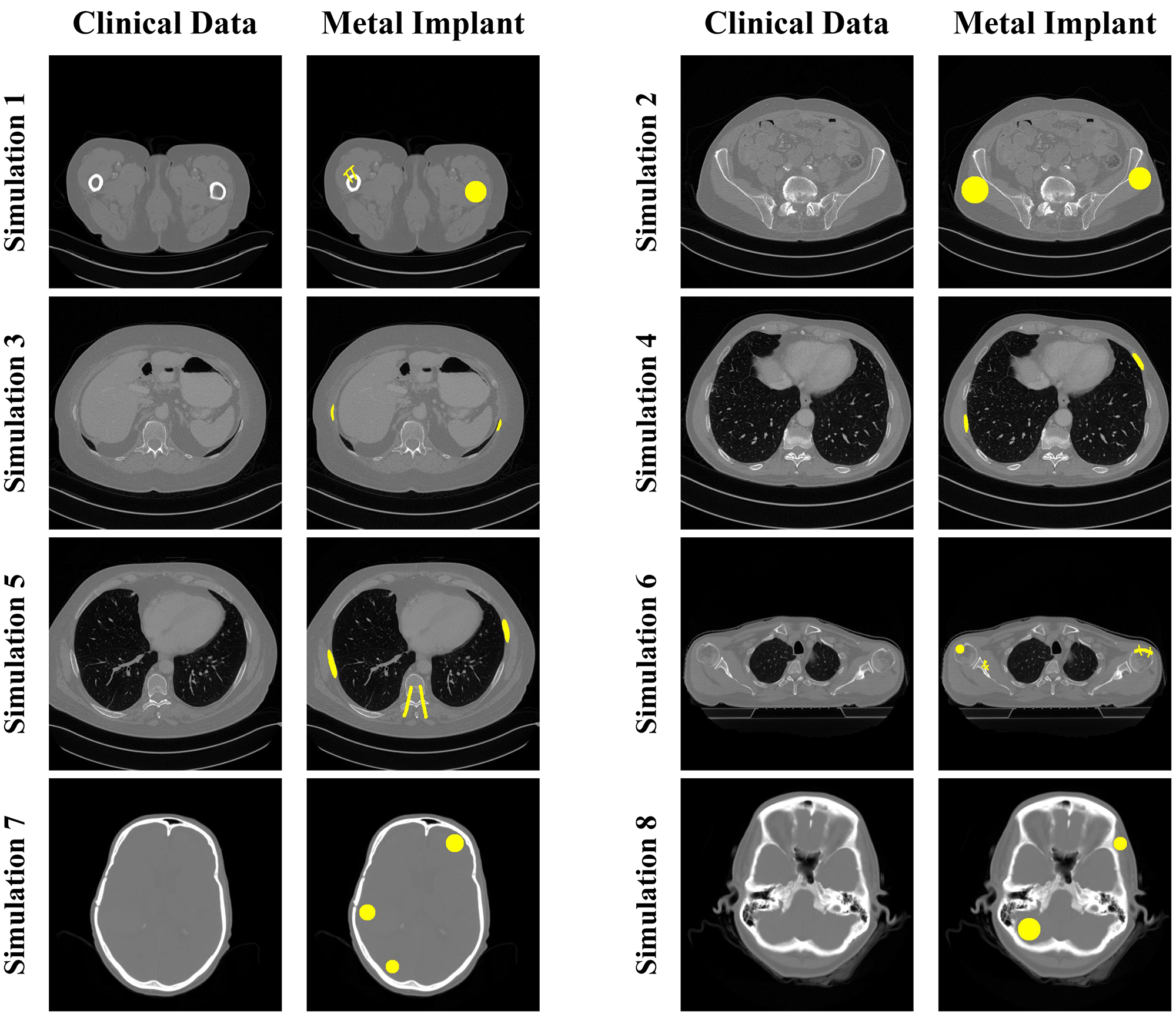

Supplement: S1 Fig — Representative images of additional simulations. Original clinical and metal implant images. Display window width/window level = 2500HU/250HU. (TIF) [file pone.0227656.s002.tif]

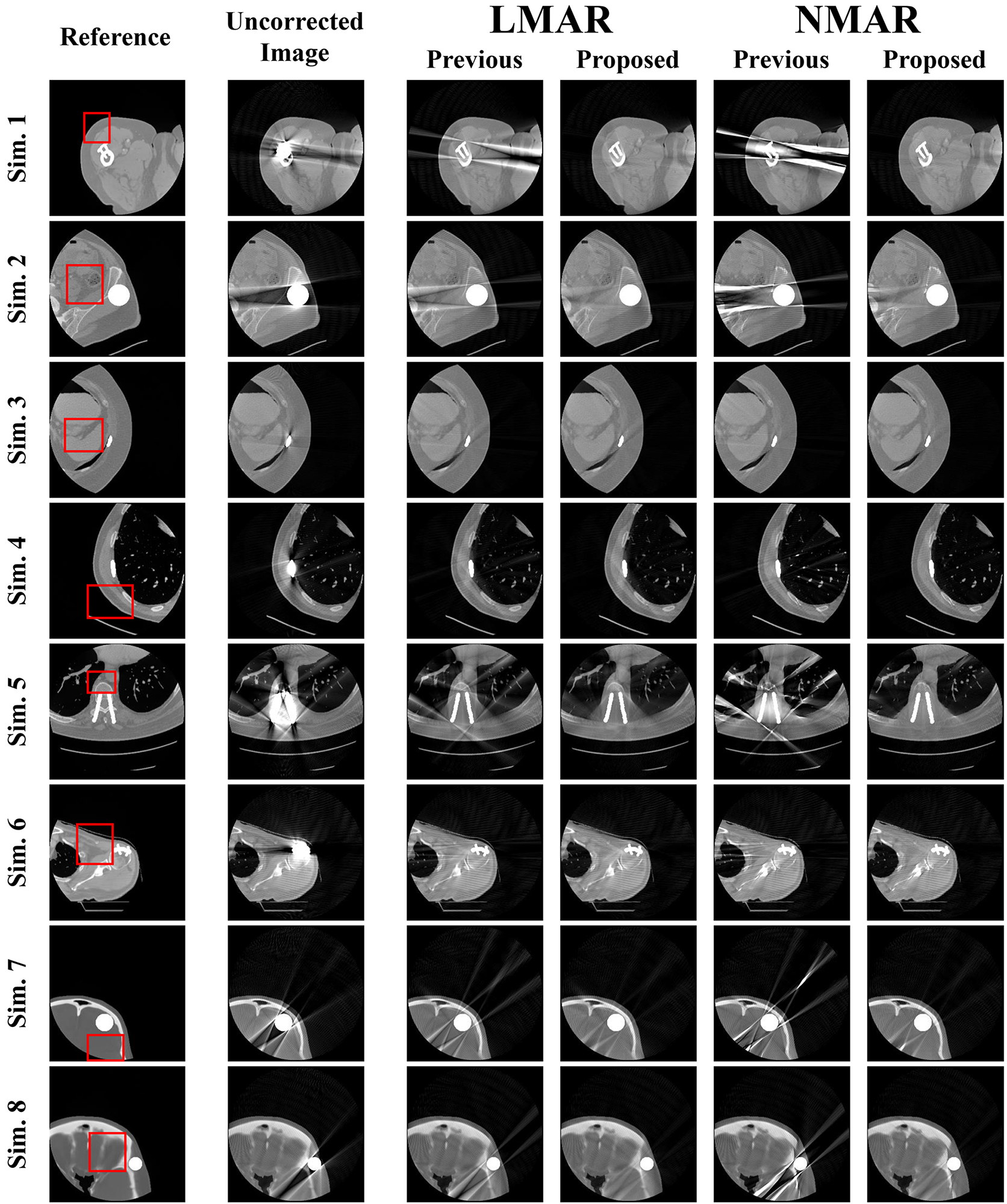

Supplement: S2 Fig — Results with additional clinical data simulation. Red boxes are the ROI of each case. Display window width/window level = 2500HU/250HU. (TIF) [file pone.0227656.s003.tif]
