# Supplementary material for: A metal artifact reduction method for small field of view CT imaging
Source: PLoS One. 2021 Jan 14;16(1):e0227656. doi: 10.1371/journal.pone.0227656 (PMC7808647; doi:10.1371/journal.pone.0227656)
Supplement: S1 Table — Quantitative evaluations of the additional clinical data simulations. NMSE and SSIM for each ROI. (DOCX) [file pone.0227656.s004.docx]

S1 Table. Supplementary table for NMSE and SSIM experiments: Quantitative evaluations of the additional clinical data simulations. NMSE and SSIM for each ROI region.

|  | | NMSE | | | SSIM | | | |
| --- | --- | --- | --- | --- | --- | --- | --- | --- |
|  |  | Previous |  | Proposed | Previous |  | | Proposed |
| Simulation 1 | LMAR | 0.1011 | 0.0481 | | 0.5566 | | 0.6316 | |
|  | NMAR | 0.2132 | 0.0358 | | 0.5113 | | 0.6798 | |
| Simulation 2 | LMAR | 0.0364 | 0.0120 | | 0.4531 | | 0.6107 | |
|  | NMAR | 0.4223 | 0.0155 | | 0.2742 | | 0.5815 | |
| Simulation 3 | LMAR | 0.0017 | 0.0014 | | 0.8240 | | 0.8397 | |
|  | NMAR | 0.0017 | 0.0014 | | 0.8271 | | 0.8416 | |
| Simulation 4 | LMAR | 0.0501 | 0.0471 | | 0.7754 | | 0.7838 | |
|  | NMAR | 0.0460 | 0.0409 | | 0.8247 | | 0.8516 | |
| Simulation 5 | LMAR | 0.0244 | 0.0215 | | 0.7989 | | 0.8337 | |
|  | NMAR | 0.1009 | 0.0146 | | 0.5850 | | 0.8494 | |
| Simulation 6 | LMAR | 0.0441 | 0.0388 | | 0.4852 | | 0.5497 | |
|  | NMAR | 0.0507 | 0.0317 | | 0.5377 | | 0.6139 | |
| Simulation 7 | LMAR | 0.0387 | 0.0357 | | 0.5340 | | 0.5993 | |
|  | NMAR | 0.0482 | 0.0303 | | 0.5699 | | 0.6192 | |
| Simulation 8 | LMAR | 0.0245 | 0.0146 | | 0.5830 | | 0.6740 | |
|  | NMAR | 0.1514 | 0.0149 | | 0.5884 | | 0.6687 | |
